# Views about clinical practice guidelines of the Indian Psychiatric Society: A survey of psychiatrists in India

**DOI:** 10.4103/0019-5545.49453

**Published:** 2009

**Authors:** Sandeep Grover, Ajit Avasthi

**Affiliations:** Department of Psychiatry, Postgraduate Institute of Medical Education and Research, Chandigarh - 160 012, India

**Keywords:** Survey, clinical practice guidelines, Indian Psychiatric Society

## Abstract

**Background:**

The Indian Psychiatric Society (IPS) constituted a task force on clinical practice guidelines (CPGs) in 2004 to formulate guidelines for management of various psychiatric disorders in the Indian setting. Over the next 4 years (2005-2008), the task force published 4 volumes of guidelines covering most of the psychiatric disorders and issues in special populations. However, till now, nothing is known about the usefulness, awareness and their implementation. This was a preliminary survey to know about the usefulness and awareness of the CPGs of the IPS.

**Materials and Methods:**

An email survey was sent to 1100 psychiatrists, of which 107 responded.

**Results:**

Only half of the responders were aware about all the 4 volumes of the guidelines and only 12.7% of the responders had read all the four volumes. About two-thirds of the responders had referred to these guidelines in their clinical practice, either occasionally (46.1%), often (16.7%) or always (2%). Similarly, more than two-thirds of the responders considered these guidelines to be helpful in making day-to-day clinical decisions in their practice, either occasionally (48%), often (19.6%) or always (3.9%). In the open-ended questions, many of the responders discussed their dissatisfaction with these guidelines and gave suggestions as to how these guidelines could be improved.

**Conclusion:**

There is need for better dissemination of the guidelines and making recommendations that can be applied in an Indian setting.

## INTRODUCTION

Over the last 50 years, there has been a tremendous advancement in the understanding of efficacy and effectiveness of various treatments in psychiatry. Hence, it is important to incorporate this new knowledge into the daily practice of every psychiatrist. One such approach is development of clinical practice guidelines (CPGs) by various national and international organizations. CPGs are “systematically developed statements to assist practitioner and patient decisions about appropriate health care for specific clinical circumstances.”[1] Clinical guidelines are expected to improve the quality of clinical decisions as they offer explicit recommendations for clinicians. Further, it is considered that CPGs can overturn the beliefs of doctors accustomed to outdated practices, improve the consistency of care and provide authoritative recommendations that reassure practitioners about the appropriateness of their treatment policies.[2]

The Indian Psychiatric Society (IPS) constituted a Task Force on CPGs in 2004 to formulate guidelines for management of various psychiatric disorders in an Indian setting. The Task Force identified psychiatrists from different parts of the country to formulate guidelines on specific conditions. For each guideline, a lead author was identified and was asked to formulate the draft of the guideline on the assigned topic along with the help of other psychiatrist(s) of his choice. Once the drafts were prepared, these were discussed in the Task Force meetings at Jaipur every year. The draft was initially read by a group of 5-9 psychiatrists, who gave their inputs. Later, the revised draft was presented to a group of 50-60 psychiatrists, who gave their comments, which were incorporated. A modified draft was supposed to be put up on the website for 4-6 weeks for everyone to comment; however, this did not happen consistently. The draft of the first volume[3] was sent to all the members by the Editor of the *Indian Journal of Psychiatry* for their comments. All the comments received were securitized by the Chairman and Convenor of the Task Force and, wherever it was felt necessary, guidelines were modified in consultation with the authors.

The members who participated in various Task Force meetings included psychiatrists from various teaching institutes (both faculty members and trainee psychiatrists), psychiatrists working with non-government organizations (NGOs) and those in private practice. From the years 2005 to 2008, the Task Force published 4 volumes of guidelines, covering most of the psychiatric disorders.[3–6] These guidelines were circulated by the Treasurer's office to various members of the society.

Although these guidelines have been published for the last 4 years, little is known about their usefulness. Hence, we carried out this survey to know the usefulness and awareness about the CPGs of IPS.

## MATERIALS AND METHODS

For this study, a questionnaire consisting of 16 questions was designed to collect information from psychiatrists practicing in India. The questionnaire included some basic questions about the psychiatrist, viz., their age, place of work, position at work, number of years of standing in psychiatry (including the training period) and whether they were involved in the development process of CPGs of IPS in any way (author in any of the guidelines, member of the expert group of the Task Force for any of the guidelines). The specific questions about the guidelines enquired their awareness about the existence of guidelines of IPS for various disorders, whether they have received the hard copies of the same, how many volumes they have read, do they refer to the CPGs of IPS in their clinical practice, do they consider these CPGs to be helpful in making day-to-day clinical decisions in their practice, do they use these guidelines as an educational tool and how they rate the content (information/evidence base) of the CPGs of IPS. In addition, 2 questions were asked regarding the other practice guidelines (e.g., American Psychiatric Society Guidelines, NICE Guidelines, etc.) and how frequently the psychiatrist follows them. Toward the end, in 2 open-ended questions, the psychiatrists were asked to give their views about the CPGs of IPS and give suggestions as to how these can be improved.

This questionnaire was sent to 1100 psychiatrists whose email addresses were available, by one of the authors (SG) during the months of October and November 2008. The psychiatrists were informed that it is a survey and it was presumed that the psychiatrists who would respond would indirectly consent for the same. The questionnaire was sent 1-6 times varying on the response and, in total, 107 responses were received, of which 5 responses were of psychiatrists of Indian origin practicing outside India for varying duration and were not at all aware of these guidelines. Hence, their data were not used in the final analysis.

## RESULTS [TABLE 1]

**Table 1:** Response of psychiatrists to the survey questions

	n (%)/mean ± SD
Age in years	43.30 ± 11.99 (range: 25-77)
Gender	
Male	81 (79.4)
Female	21 (20.6)
Place of work	
Academic institute	53 (52)
Predominantly an academic institute/medical college with part-time private practice	11 (10.8)
Private practice alone	38 (37.3)
Position at work	
Faculty member in an academic institute/medical college	38 (37.3)
Consultant in a private hospital/NGO	42 (41.2)
Senior resident	12 (11.8)
Trainee resident (MD/DNB/DPM students)	09 (8.8)
Retired	01 (1.0)
Number of years of standing in psychiatry (including the training period)	17.58 ± 11.33 (range: 1.5-45)
Were you involved in the development process of the Clinical practice guidelines of the Indian	
Psychiatric Society in any way (author in any of the guidelines, member of the expert group of the task force for any of the guidelines)	
Yes	24 (23.5)
No	78 (76.5)
Are you aware that the Indian Psychiatric Society is publishing guidelines for various disorders since the last 4 years	
Aware about all the 4 volumes	52 (51)
Aware about 2-3 volumes	25 (24.5)
Aware about 1 volume	12 (11.8)
Not aware at all	13 (12.7)
Have you received the copies (hard copies)	
Yes, all 4 volumes	23 (22.5)
Only 3 volumes	12 (11.8)
Only 2 volumes	20 (19.6)
Only 1 volume	19 (18.6)
None	28 (27.5)
How many volumes have you read?	
All 4 volumes	13 (12.7)
3 volumes	06 (5.9)
2 volumes	18 (17.6)
1 volume	12 (11.8)
Some guidelines from one or more volumes	33 (32.4)
None	20 (19.6)
Have you ever referred to the clinical practice guidelines of the IPS in your clinical practice	
Always	02 (2.0)
Often	17 (16.7)
Occasionally	47 (46.1)
Never	19 (18.6)
Not applicable*	16 (15.7)
Do you think that the clinical practice guidelines of IPS are helpful in making day to day clinical decisions in your practice#	
Always	04 (3.9)
Often	20 (19.6)
Occasionally	49 (48.0)
Never	10 (9.8)
Not applicable*	19 (18.6)
Have you used the clinical practice guidelines of IPS as an educational tool	
Always	05 (4.9)
Often	21 (20.6)
Occasionally	30 (29.4)
Never	27 (26.5)
Not applicable*	19 (18.6)
How do you rate the content (information/evidence base) of the clinical practice guidelines of the Indian Psychiatric Society#	
Good	28 (27.5)
Average	40 (39.2)
Poor	13 (12.7)
Not applicable*	19 (18.6)
No comments@	01 (1.0)
Are you aware of any other clinical practice guidelines (e.g., American Psychiatric Society guidelines, NICE Guidelines, etc.)#	
Yes	99 (97.1)
No	02 (02.0)
How commonly do you rely on the clinical practice guidelines of other societies (e.g., American Psychiatric Society Guidelines, NICE Guidelines, etc.)	
Always	15 (14.7)
Often	42 (41.2)
Occasionally	36 (35.3)
Never	09 (8.8)

* Not applicable: if you do not have access to any one of them or you are not aware of them

# 1 response was missing for each of these questions

@ One of the responders added the response as “No comments.”

### Response rate

The response rate of our survey was poor (9.72%).

### Profile of responders

The mean age of the psychiatrists who responded was 43.3 years. In terms of age group, about 45% of the responders were less than 40 years of age and another 45% were aged between 41 and 60 years. A majority of them were male (79.4%). More than half of the responders were working in academic institutes at various levels (faculty, senior resident and junior resident), and more than one-third of them (37.3) were into full-time private practice. In terms of position at work, more than 40% were consultants in a private hospital or an NGO and more than one-third were in a faculty position (37.3) in institutes. About 20% of the responders were working as Residents (Senior/Junior). The mean number of years of standing in psychiatry of the responders was 17.58 years. Only one-fourth of the responders were involved in formulation of guidelines, either as authors or as members of various task forces.

### Awareness about and access to CPGs of IPS

Only half of the responders were aware about all the 4 volumes of the guidelines. One-fourth of them were aware of 2-3 volumes and 12.7% of the responders were not at all aware of the existence of CPGs of IPS. However, less than one-fourth of the responders had received (22.5%) all the four volumes of the CPGs and slightly more than one-fourth of them (27.5%) had not received any of the four volumes of CPGs.

### Use of CPGs of IPS

Only 12.7% of the responders had read all the four volumes of CPGs. Nearly one-third of the responders had read *“some guidelines from one or more volumes”* and 20% of them had not read any of these guidelines. However, about two-third of the responders had referred to these guidelines for their clinical practice, either occasionally (46.1%), often (16.7%) or always (2%). Similarly, more than two-thirds of the responders considered CPGs of IPS to be helpful in making day-to-day clinical decisions in their practice, either occasionally (48%), often (19.6%) or always (3.9%). Further, more than half of the responders used the CPGs of IPS as an educational tool, either occasionally (29.4 %), often (20.6%) or always (4.9%).

### Content of CPGs of IPS

The most common response was average (39.2%), followed by good (27.5%) and poor (12.7%).

### Awareness and use of other guidelines

Almost all were aware about the existence of guidelines of other societies and these guidelines were often, occasionally and always used by 41.2%, 35.3% and 14.7% of the responders respectively.

### Comparison between those involved and those not involved in formulation of CPGs

When we compared the responses of those who were involved in formulation of CPGs (*n* = 24) and those who were not involved, it was seen that those who were involved in the formulation of guidelines were more aware of all the volumes (Fischer exact value - 12.31**, *P* = 0.006) and had more frequently received copies of one or more volumes (Fischer exact value - 10.73*, *P* = 0.03). There was no significant difference in response to other questions.

### Views about the CPGs of IPS

The response to the open-ended questions varied. Some of the responders left the questions unanswered. Most of the responders gave the response in a few words and some of the common themes that emerged from these responses were “a good beginning/attempt,” “not uniform,” “replica/amalgam of current available international guidelines,” “not very useful” and “no comment.”

However, some of the responders gave details of their satisfaction or dissatisfaction with the CPGs of IPS. We would discuss the common themes of these. Regarding the CPGs of IPS, one of the residents wrote that *“It is a good endeavor, because it helps us share experiences of senior psychiatrists and plan treatment accordingly for Indian patients.”* Some of the responders wrote that the guidelines take some of the cultural factors into consideration and are hence helpful. Another common theme that emerged from the responses was that the guidelines should be *“updated periodically.”*

There were many themes with regard to dissatisfaction. First, many of the responders were dissatisfied with the guidelines because the guidelines did not include Indian data or did not address to the situations that are faced specifically in Indian situations. However, some of the responders also acknowledged that there is lack of Indian data on some of the issues. One of the responders expressed that *“I don't think that the guidelines are likely to be evidence-based, as there is a serious dearth of Indian literature on matters that clinically matter. The current guidelines are either based on research done outside or impressionistic (not evidence based). The guidelines are also too ideal to be applicable in the real world - they are applicable to only a miniscule proportion of patients, because of myriad practical problems. The guidelines should be made in hierarchical fashion, to be applicable for primary, secondary and tertiary care centers, but there is no evidence base for this.”* Another responder commented *“They have nothing new to say. One gets an impression that they have been written taking few things from various guidelines. Guidelines have to be locally relevant. They are like Indian adaptations of international guidelines. The real problem is that there is no good data from controlled studies involving large samples of Indian patients. Therefore, guidelines end up saying what other guidelines recommend. If you have nothing new to say, there is no point in wasting time to develop guidelines.”* Still another responder wrote *“The guidelines appear to be put together based on those from the West. Nothing local or Indian about them and not based on any Indian evidence. The guidelines appear to be put together by members who may not have had practical experience with the specialty area being written about. There is no legal or societal mandate to follow these guidelines.”* A responder suggested that *“there should be clinical trials in the different parts of the country in different disorders with strict methodology.”*

A second common theme of dissatisfaction that emerged was that these guidelines were not user-friendly. Some of the responders wrote that these guidelines are *“lengthy,” “overinclusive,” “should be more practical,” “not private practice based,” “need to be more concise, focusing more on local cultural issues and day to day problems in patient management,” “simplify and to make it point by point presentation such that the practitioners, postgraduates and clinicians can benefit by them in their day to day practice,” “more user friendly format - like the algorithm for treatment of depression” and “quality of printing and typographical error in the document are the weak points.”* The third common theme that emerged was that these guidelines were not uniform. One of the responders wrote that *“most IPS CPGs are not clinical practice guidelines in the true sense”* and another commented that they *“should be standardized on the lines of APA and NICE guidelines.”* Further, one of the responder wrote that *“It's just a compilation of the literature on related topics, sometime not even providing any guideline, leaving the reader in a chaos.”* The fourth common cause of dissatisfaction was that the guidelines did not take all the available evidence into consideration while formulation. One of the responder wrote that *“evidence base has to be improved.”* Further, one of the responders expressed dissatisfaction on the way the guidelines were formulated and wrote that *“guidelines preparation are very specialized bits of work and ours do not follow the international norms for guideline preparation.”*

### How to improve

Again, some of the common themes emerged as to how the CPGs of IPS can be improved. The responses covered various areas like how to generate data and go about the formulation of guidelines, who should be part of the formulation of guidelines, how the guidelines should be presented and how to improve their circulation and utility.

Regarding how to generate data and go about the formulation of guidelines, some of the responders expressed that there is need to conduct national-level studies with sound methdology before formulating the guidelines, whereas others expressed that expert consensus guidelines can be formulated and, if required, can be field tested before they are adopted. One of the responder opined that *“Guidelines should be made locally applicable and useable from local evidence. Task force (of professionals both from academic and private practice) in each area should be created for framing guidelines. The guidelines so framed must be circulated for consensus and applicability among larger body of professionals working in the specific area. If possible the guidelines should be field tested to see the usefulness and applicability in routine clinical practice.”* Another responder expressed that the IPS can *“take a detailed survey with questionnaires with leading practioners and try to find what guidelines each has developed on his/her experience which has shown good result in their clinical practice, then try to match with the theoretical academic model to find the truth why they are more useful.”* However, one of the psychiatrists observed that *“rather than formulating guidelines for everything, IPS should focus on few areas - there is little India-centered research to inform guidelines, also there are few experts who work exclusively in specific disorders, so the IPS should reconsider its approach to development of CPGs. It would be helpful if so many CPGs are not developed at one time, rather CPGs should be developed in fields that are mature enough for their development in India. Most western associations do not have guidelines for all disorders. May be we should stop aping the West and the IPS should think in terms of broader groupings like severe mental illness and common mental disorders etc. (to be able to gather enough genuine experts) or start by forming taskforces that can formulate directions for relevant research before coming to the issue of CPGs.”*

With respect to “who should be involved in the formulation,” a common theme emerged that the Task Force should include more academicians, practitioners and advocacy groups and should formulate guidelines on the basis of expert opinion. However, some of the psychiatrists were of the view that *“only professors with established reputation and who have done commendable work in that particular area should be asked to prepare guidelines.”*

In terms of presentation, as discussed in the earlier section, one of the common opinions was that the guidelines must be more uniform. Other common themes were the printing mistakes. One of the psychiatrists wrote *“I think that more attention should be given to eliminate printing mistakes, a more visually pleasing formatting and a better quality of the hard copies should be made available. One has to look at Western guidelines to see the superior looks and quality they have.”*

Another important aspect was the circulation of guidelines. Many psychiatrists expressed that the IPS should mail the hard copies to all in time and the guidelines must be put on the website, from where they can be easily accessed and the readers can also express their views on the website. Some of the psychiatrists expressed that the IPS should discuss the guidelines in conferences and continuing medication education programmes.

Last but not the least, many psychiatrists expressed that this good endeavor of the IPS should continue in formulating the guidelines and the guidelines must be updated regularly.

## DISCUSSION

This is the first survey that assessed the views of Indian psychiatrists about the guidelines published by the IPS. The survey was emailed by one of the authors (SG), who has been a coauthor in 3 of the IPS guidelines but did not hold any office of the IPS. The survey was deliberately not sent by the other author of this article (AA), Convenor of the Task Force for formulation of guidelines, as it was considered that if his identity was disclosed as a member of the survey team, it may influence the responses.

Although the survey was not sent to all the IPS members, it covered about one-third membership of the society. However, the response rate was poor compared with one of the previous surveys conducted on the practice of electroconvulsive therapy in India.[7] However, in that survey, the head of the institutes or department were approached rather than individual psychiatrists. The response rate to our survey was better than the response rate to a survey in the West in which the authors assessed the extent to which guidelines are used in the treatment of bipolar disorder[8] but much worse than the survey of Rees *et al*,[9] which evaluated the attitude of psychiatrists toward evidence-based guidelines.

The relatively poor response rate in the current survey can be due to various reasons. First, the survey was sent by one of the psychiatrist who was not very well known among the senior psychiatrists. Another reason for the poor response could be that many of the psychiatrists are themselves not computer savvy, the way the survey was performed (although it had multiple choice questions, the responders were required to modify the word document themselves) and lack of understanding in general about the importance and purpose of such a survey.

Although the response rate was poor, there was a fair representation of psychiatrists of different age groups, place and position of work and number of years in psychiatry. Only one-fourth of the responders were involved in the formulation of guidelines, either as authors or as members of various Task Forces. Further, there was no statistically significant difference between those involved and those not involved in formulation on all the multiple choice questions except for being aware about more number of volumes and receiving more number of volumes. This suggests that the responses were not influenced by the participation in the formulation of the guidelines.

Only 51% of the responders were aware about all the 4 volumes and only 22.5% of the responders had received these. This suggests that although IPS has taken an initiative in the formulation of these guidelines, they were not being disseminated properly. Hence, the IPS should make efforts to increase the awareness about the presence of such guidelines and they should be circulated to the membership. As suggested by some of the responders, the guidelines can be put on the IPS website and the guidelines can be discussed in some of the focused CME programmes to improve the dissemination of the guidelines.

With respect to the use of the IPS guidelines, as is evident from the responses, at least two-thirds of the responders used them at least occasionally in their clinical practice and considered these to be helpful at least occasionally. Similarly, more than half of the responders used these guidelines at least occasionally as an educational tool, while two-thirds of the psychiatrists rated these guidelines as average or good. Although these responses may not appear great, we think that it reflects that at least some of the psychiatrists are interested in having Indian CPGs and are willing to follow them. In the survey of psychiatrists regarding the extent to which guidelines are used in the treatment of bipolar disorder in the West, 64.1% of the responders reported making routine use of treatment guidelines while taking clinical decisions.[8] On comparing the findings, it appears that the CPGs of IPS are not followed to the same extent as is done in the West.[9]

Studies carried out on guidelines for various physical and psychiatric disorders in the West have shown that guidelines that are easy to understand, can easily be tried out and do not require specific resources have a greater chance of being used.[10–14] Based on this and the responses of the psychiatrists in this survey, in future, such recommendations should be made in the IPS guidelines, which are easy to understand and can be easily tried out.

However, numerous studies have demonstrated that despite the publication and dissemination of a large number of guidelines, they always do not lead to a meaningful change in day-to-day clinical practice.[15–17] This kind of failure may occur because clinicians are unaware of new guidelines, do not familiarize themselves with guideline content, do not agree with treatment recommendations or do not work in settings with adequate logistical support for implementation.[18] This suggests that just publishing and dissemination of guidelines may not be sufficient to have a change at the practical level. Hence, the IPS should discuss these guidelines at different levels and try to understand the concerns of the stakeholders before publishing it.

The views to the open-ended questions of this survey can be a good starting point from where we can reflect and plan ahead. In future, the IPS can take up some of the suggestions while formulating the revision of guidelines. According to our view, at least the following suggestions should be incorporated:

- Formulate guidelines on only 1 topic at a time.
- A uniform pattern must be followed in writing and compiling the evidence for all the guidelines.
- The expert groups should be bigger.
- Besides the academicians and practitioners, the expert group should comprise of subjects from advocacy groups and representatives of patients.
- The initial draft of guidelines can be presented in the zonal- and state-level conferences by members of the expert group to seek the opinion of various members.
- Carry out field trials at least in 1-2 centers before recommending a particular prescription. If not feasible, an expert consensus should be made, which can be arrived at by sending questionnaires to randomly selected members.
- The recommendations based on data available from other countries can be graded based on the level of evidence.
- The recommendations about the non-pharmacological management can be graded as to what minimum can be done when specific therapies cannot be carried out.
- Issues specific to the Indian context, on the basis of expert consensus can be incorporated.
- Usefulness of traditional methods like yoga and pranayam in the management of various psychiatric disorders can be arrived at by carrying out surveys and the findings can be incorporated in the guidelines.
- Proper dissemination of guidelines for soliciting suggestions before accepting the document.
- Proper dissemination of guidelines after publishing the same. It can be published as a supplement to the *Indian Journal of Psychiatry*.
- Guidelines should be updated periodically.
- The guidelines should be evaluated on an AGREE instrument.[19]

### Limitations

It is important to consider the limitations of this survey. It included the opinion of only 102 psychiatrists. Considering that there are about 3500 psychiatrists in India, this survey does not reflect the overall opinion of all the psychiatrists in India. In future, a larger survey should be conducted covering a greater number of members of the society.
